# Anomalous Origins of the Left Vertebral and Left Gastric Arteries in the Same Cadaver: Case Report

**DOI:** 10.7759/cureus.87129

**Published:** 2025-07-01

**Authors:** Yasmine Gharbieh, Aftab Shaik, Grant Barber, George Prada

**Affiliations:** 1 Department of Anatomical Sciences, Sam Houston State University College of Osteopathic Medicine, Conroe, USA; 2 Department of Clinical Anatomy, Sam Houston State University College of Osteopathic Medicine, Conroe, USA

**Keywords:** anomalous origin of the left vertebral artery, aortic arch branches, left gastric artery, left vertebral artery variation, vertebral artery anomalies

## Abstract

Variations in the aortic arch and abdominal aorta possess important clinical implications that are frequently overlooked or underestimated, often being identified incidentally during surgical interventions or post-mortem examinations. This case report describes the rare concurrent occurrence of two such anomalies: an aberrant origin of the left vertebral artery and an anomalous origin of the left gastric artery. These findings were made during routine anatomical dissection of a 77-year-old white female cadaver at the Clinical Anatomy Laboratory of Sam Houston State University College of Osteopathic Medicine. The donor’s cause of death was reported as metastatic bone cancer. The aberrant left vertebral artery was observed arising directly from the aortic arch, positioned between the left common carotid and left subclavian arteries. The anomalous left gastric artery originated directly from the abdominal aorta, forming a separate trunk lateral to the celiac trunk, rather than branching from it. Furthermore, the right and left inferior phrenic arteries were observed to originate from this aberrant trunk of the left gastric artery rather than directly from the abdominal aorta.

These vascular anomalies were discovered incidentally during the dissection and analysis of eight cadavers, as part of a study aiming to compare the distances between the main branches of the thoracic and abdominal aorta. This report examines the anomalous branching patterns found on the aortic arch and abdominal aorta, with a detailed review of relevant anatomical and embryological literature. High-resolution images captured during dissection provide clear visual documentation of these vascular variations, offering a level of detail often lacking in existing publications. Understanding such anomalies is critical not only for anatomists but also for clinicians and surgeons involved in abdominal and thoracic procedures, as failure to recognize these variants may result in surgical complications or misinterpretation of imaging studies.

## Introduction

The architecture of the aortic arch, along with its primary branches, follows a well-established pattern that serves as a fundamental reference for surgical procedures and cadaver-based anatomy education for medical students. The standard anatomical configuration includes three major branches emerging from the aortic arch: the brachiocephalic trunk, the left common carotid artery, and the left subclavian artery [1]. The vertebral arteries typically arise as the first branches from their respective subclavian arteries. Similarly, the celiac trunk's classical trifurcation into the left gastric, splenic, and common hepatic arteries is a key landmark in abdominal vascular surgery [1]. However, the rare concurrent presentation of the left vertebral artery originating directly from the aortic arch, positioned between the left common carotid and left subclavian arteries, as well as the independent origin of the left gastric artery (LGA) from the abdominal aorta, represent significant deviations from these traditionally observed patterns.

The aberrant origins of the vertebral and gastric arteries are believed to result from the overabsorption of embryonic tissue, developmental failure in the anastomosis between arteries, and the failure of normal regression during vascular development [2]. Unusual branching patterns of the left vertebral artery (LVA) have been classified into three types (A, B, and C) based on their origin [3]. In Type A, the LVA arises directly from the aortic arch, positioned between the left common carotid artery (LCCA) and the left subclavian artery (LSA), which is the most common variant. Type B involves the LVA originating from a common trunk formed by the brachiocephalic trunk (BCT) and LCCA, rather than arising separately. In Type C, the LVA originates from the aortic arch proximal to the origin of the LSA [4].

In this case study, we report rare variations in the branching of the LVA and the LGA, both occurring in the same donor. This case is also distinctive in that the vascular anomalies of the LVA and the LGA are clearly visualized in the cadaveric specimen, allowing for direct observation of their arterial origins and courses. In our case, the vascular anomaly corresponds to a Type A variant pattern, characterized by LVA originating directly from the aortic arch between the left common carotid and left subclavian arteries. This variant has been reported to occur with a prevalence ranging from 0.79% to 8% [3]. Additionally, an anatomical variation was observed in which the LGA originated directly from the abdominal aorta. This anomaly is thought to result from atypical embryological development of the ventral splanchnic arteries, which can give rise to significant deviations in vascular branching patterns. The reported prevalence of this variation ranges from 0.5% to 15% [5].

Anatomical variations of the aortic arch and descending aorta, though relatively rare, are well documented in the literature and are often asymptomatic. As a result, many individuals may remain unaware of these vascular anomalies throughout their lives. However, comprehensive knowledge and precise visualization of such variations (particularly within the aortic arch and abdominal aorta) are essential for the accurate planning and safe execution of vascular interventions and surgical procedures involving the chest, neck, and abdomen. Unrecognized anatomical deviations can lead to intraoperative complications, including catheterization challenges, unexpected bleeding, or misinterpretation of imaging studies. In particular, variations in vertebral artery origin and course may pose additional risks during aortic or gastric procedures. Therefore, preoperative recognition of these anomalies is critical to minimizing surgical risks and optimizing procedural outcomes.

## Case presentation

**Figure 1:**
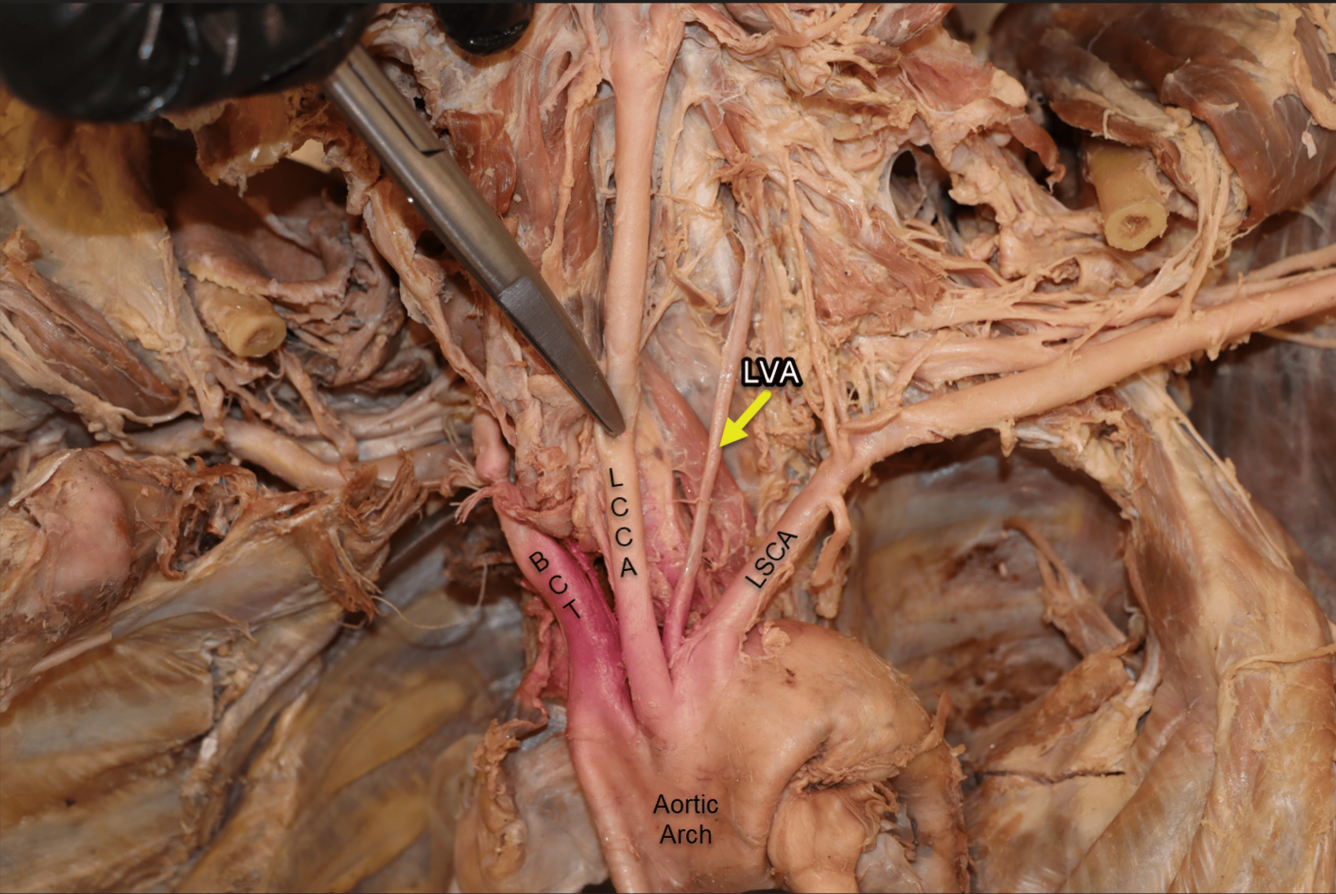
Anomalous vertebral artery. The left vertebral artery (yellow arrow) demonstrates an anomalous origin, arising directly from the aortic arch (AA) rather than from the left subclavian artery (LSCA). It ascends posteriorly, situated between the left common carotid artery (LCCA) and the LSCA. In anatomical position (viewed right to left), the brachiocephalic trunk (BCT) is identified as the first branch of the aortic arch (AA).

The most common variation in the origin of LVA is its emergence directly from the aortic arch, rather than the LSA. This anatomical anomaly of the LVA (Figure 1) was identified in a 77-year-old female cadaver during an exploratory dissection conducted to measure the length and diameter of the aortic arch branches. In this case, the LVA originated directly from the aortic arch, positioned slightly posterior between the LCCA and the left subclavian artery (LSCA).

**Figure 2:**
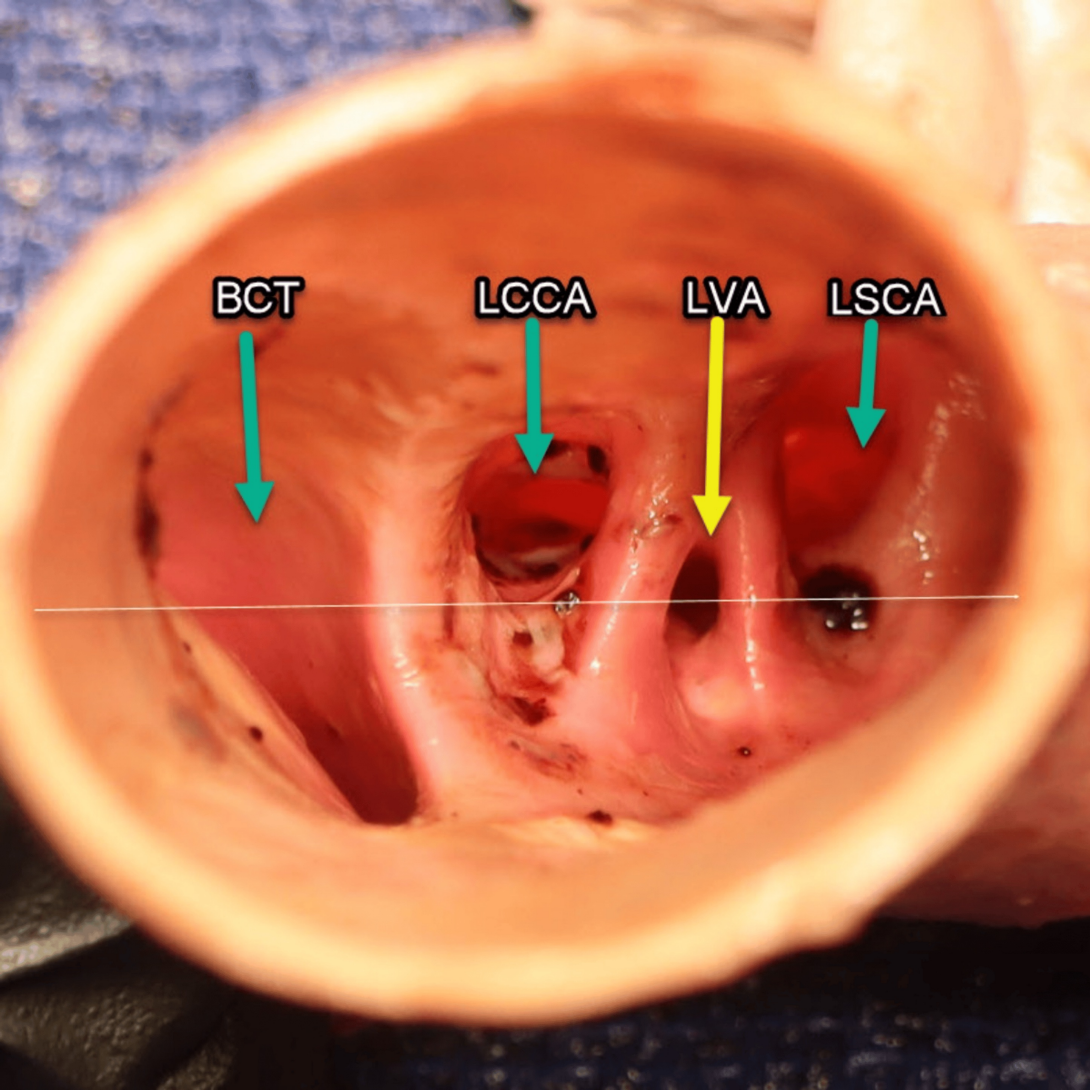
Aortic arch transverse section. An inferior view of the transverse section of the aortic arch revealed four orifices from left to right (reader view): the brachiocephalic trunk (BCT), the left common carotid artery (LCCA), the left vertebral artery (LVA, yellow arrow), and the left subclavian artery (LSCA). A white hypothetical reference line is projected through the midpoint of the left vertebral artery (LVA) origin at the level of the aortic arch, providing a basis for comparison with the three other arteries arising from the aortic arch.

A transverse section of the aortic arch (Figure 2) was performed to observe the four arterial orifices, corresponding to the in situ cadaveric findings of the brachiocephalic trunk (BCT), LCCA, LVA, and LSCA. Blood clots were meticulously removed from both the aortic arch and the orifices of these arteries. Subsequently, the specimen was thoroughly irrigated with a cadaveric solution to enhance the visibility of vascular structures and facilitate the identification of any anomalies. This step was essential for clearly visualizing the unusual origin of the LVA arising directly from the aortic arch as demonstrated in the image below. When an imaginary (white) horizontal line is drawn through the transverse section of the aortic arch, passing through the midpoint of the LVA orifice, it can be observed that the LVA orifice is positioned inferior to and narrower than the orifices of the left common carotid and left subclavian arteries, but superior to the opening of the BCT.

**Figure 3:**
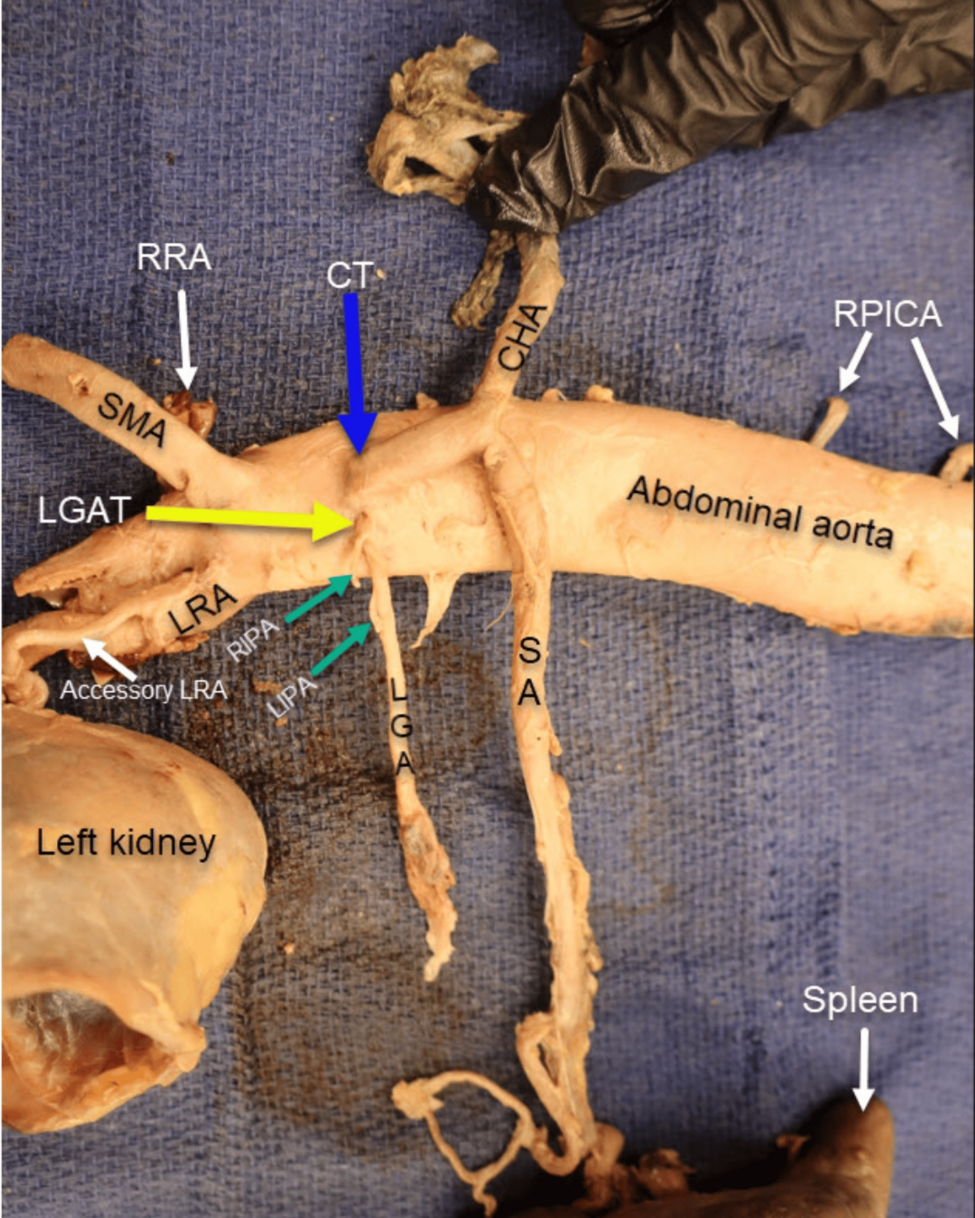
Aberrant left gastric artery trunk. The left gastric artery (LGA, yellow arrow) arises directly from the abdominal aorta (AA) as a distinct trunk. Both the right and left inferior phrenic arteries (RIPA and LIPA, green arrows) originate from this anomalous LGA trunk. The celiac trunk (CT, blue arrow) gives rise to two primary branches: the splenic artery (SA) and the common hepatic artery (CHA). Inferior to the CT, the superior mesenteric artery (SMA) emerges from the AA. The right renal artery (RRA) and left renal artery (LRA) also arise from the AA, with an accessory left renal artery observed branching from the LRA. Additionally, the right posterior intercostal arteries (RPICA) are seen originating from the thoracic aorta.

During the examination and measurement of the branches and their respective distances from the abdominal aorta (AA), a second anatomical variation (Figure 3) was identified in this cadaver: an anomalous origin of the LGA. Uniquely, the LGA arose as an independent trunk directly from the AA, positioned lateral and slightly to the left of the celiac trunk (CT). Furthermore, both the right inferior phrenic artery (RIPA) and the left inferior phrenic artery (LIPA) originated from this aberrant LGA trunk.

**Figure 4:**
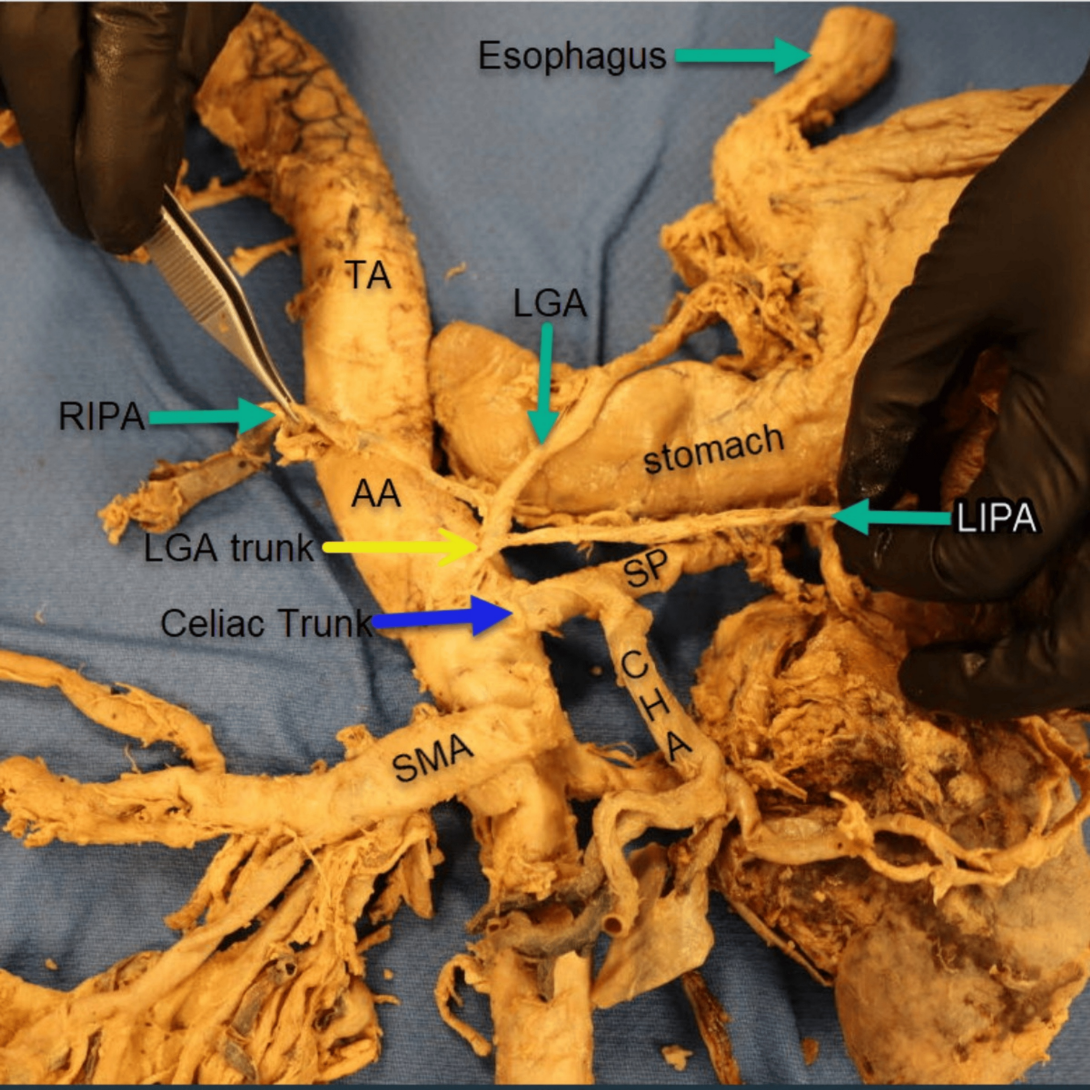
Left gastric artery trunk. The left gastric artery trunk (LGAT, yellow arrow) branches directly from the abdominal aorta (AA), positioned superiorly and lateral to the celiac trunk (CT, blue arrow). In this case, the CT gives rise to the splenic artery (SP) and the common hepatic artery (CHA). The superior mesenteric artery (SMA) originates inferior to the CT. Both the right and left inferior phrenic arteries (RIPA and LIPA) branch off the LGAT.

For comparison, an anomalous origin of the LGA was also observed in a second specimen (Figure 4), an 89-year-old male donor whose cause of death was coronary artery disease. In this case, the LGA also arose directly from the abdominal aorta as a small trunk, located superior and slightly lateral to the CT. It ascended to supply the proximal curvature of the stomach. Both the right and left inferior phrenic arteries (RIPA and LIPA) originated from this LGA trunk, with the RIPA arising superiorly and the LIPA arising inferiorly. In this donor, the CT gave rise to only two branches: the splenic artery (SA) and the common hepatic artery (CHA). The superior mesenteric artery (SMA) originated inferior to the CT.

## Discussion

The cadaveric case analyzed in this study was a 77-year-old white woman, whose documented cause of death was metastatic bone cancer. This specimen was part of a broader anatomical investigation focused on measuring the distances and diameters of the major branches of the thoracic and abdominal aorta across eight cadavers. Among these eight specimens, two vascular anomalies were identified - both within the same donor - offering a rare and valuable opportunity for comprehensive anatomical and morphological evaluation. The first was an anomalous origin of the LVA directly from the aortic arch instead of the LSCA. The second was a rare anatomical variation in which the LGA originated directly from the abdominal aorta as a trunk, rather than from the celiac trunk as typically observed. Although the precise etiologies of these vascular anomalies remain unclear, they are generally attributed to disruptions in embryological development.

In this case, the LVA was observed to originate directly from the aortic arch, positioned between the left common carotid artery and the LSCA, a recognized anatomical variation. In light of this anomaly, the diameters of both the left and right vertebral arteries were measured for comparative analysis. Given the more proximal location of the LVA on the aortic arch (where arterial pressure is generally higher), we hypothesized that the LVA would exhibit a greater diameter than the right vertebral artery (RVA), which originates from the subclavian artery. However, measurements obtained using a high-precision electronic caliper revealed that both vertebral arteries had identical diameters of 2.05 mm, regardless of their origin. This result does not support our initial hypothesis and suggests that vertebral artery diameter may be influenced by factors other than proximal arterial pressure alone.

The vertebral artery (VA) origin anomalies may be single, which represents 96% of the cases, or dual (affects both origins). For a single origin, the LVA is most often (85%) above the RVA (12%) or bilateral (3%) [2]. The commonest VA origin variant is the LVA, originating from the arch between the LCCA and the LSCA, whose prevalence is 2.5%-8.3% [6].

From an embryological perspective, the vascular variations observed in this case report can be attributed to the complex and dynamic developmental processes of the embryonic arterial system. During early embryogenesis, the aortic arches and their associated dorsal aortic segments undergo significant remodeling, regression, and anastomosis. Variations such as the LVA originating directly from the aortic arch, or the LGA arising independently from the abdominal aorta, likely result from deviations in the normal regression or persistence of the third, fourth, and sixth pairs of the primitive aortic arches [2] of embryonic vessels during this period of vascular morphogenesis. Similarly, the independent origin of the LGA from the abdominal aorta suggests a deviation from the typical development of the 13th ventral segmental artery, as normal development leads to the celiac trunk’s typical trifurcation [7]. This developmental variation highlights the intricacies of vascular embryogenesis. Alterations in the normal regression patterns can result in clinically significant anatomical variations, as demonstrated in this dissection.

The concurrent presentation of the anomalous origins of the LVA and LGA in a single donor has significant implications for both clinical practice and medical education. Clinically, awareness of such variations is crucial for vascular surgeons, especially during procedures involving the aortic arch or celiac trunk [2,8]. These variations can impact the selection and approach of catheterization techniques, as altered vessel origin angles may require customized catheter shapes and modified procedural approaches. Preoperative identification of these variations through computer tomography angiography (CTA) or magnetic resonance angiography (MRA) enables surgeons to adjust their techniques, potentially reducing procedure time and minimizing the risk of iatrogenic injury during both open and endovascular interventions [2,8]. Documenting these anomalous origins not only contributes to the literature on vascular anomalies but also underscores the importance of comprehensive preoperative vascular mapping.

Furthermore, this case serves as an invaluable teaching tool for those undergoing medical education, particularly in gross anatomy laboratories and surgical training programs [7]. By incorporating and raising awareness of vascular anatomical variations into the medical curriculum, educators can better prepare future healthcare professionals for the anatomical diversity they may encounter in clinical practice.

A key limitation of this study lies in the small and demographically limited sample size, which constrains the generalizability of the findings. In particular, the narrow distribution of vascular anomalies across age, sex, and racial or ethnic groups limits our ability to draw broader conclusions. To better understand the prevalence, developmental significance, and potential clinical implications of these anomalies, future research should involve larger, more diverse populations.

## Conclusions

This case report presents the concurrent presence of two notable vascular anomalies: a left vertebral artery originating directly from the aortic arch, and a left gastric artery arising independently from the abdominal aorta as a separate trunk. The vertebral artery variant was identified in one cadaver, whereas the left gastric artery anomaly was observed in two of the eight cadaveric specimens examined. These findings add to the expanding body of literature on vascular anatomical variations and underscore their clinical relevance. Recognizing such anomalies is essential for reducing the risk of intraoperative and procedural complications, and they serve as valuable educational references in both anatomical and surgical training. This case reinforces the importance of detailed anatomical research and its translational impact on clinical practice across diverse patient populations. The study further highlights how awareness of aberrant branching patterns in the aortic arch and abdominal aorta can contribute to improved outcomes in cardiothoracic and abdominal surgeries, thoracic radiologic interventions, and comprehensive patient care.
